# From dysbiosis to mechanisms: Why cervicovaginal microbiome-HPV studies must catch up with biology

**DOI:** 10.1371/journal.ppat.1013830

**Published:** 2025-12-30

**Authors:** Mariano A. Molina

**Affiliations:** 1 Department of Laboratory Medicine, Karolinska Institutet, ANA Futura, Huddinge, Sweden; 2 Department of Cellular Therapy and Allogeneic Stem Cell Transplantation (CAST), Karolinska University Hospital, Huddinge, Sweden; 3 Instituto de Ciencias Médicas, Las Tablas, Panama; Brown University, UNITED STATES OF AMERICA

## Introduction

Elucidating the influence of the cervicovaginal microbiome on HPV infection dynamics and the trajectory toward neoplastic change has been a persistent and important challenge in cervical cancer research [1,2]. HPV infections are common in reproductive-age women, including both low-risk and high-risk genotypes [3,4]. In this context, interactions between HPV and the cervicovaginal microbiome are not confined to carcinogenic infections but occur across a broad spectrum of transient, persistent, and clinically silent HPV states [5,6].

**Fig 1:**
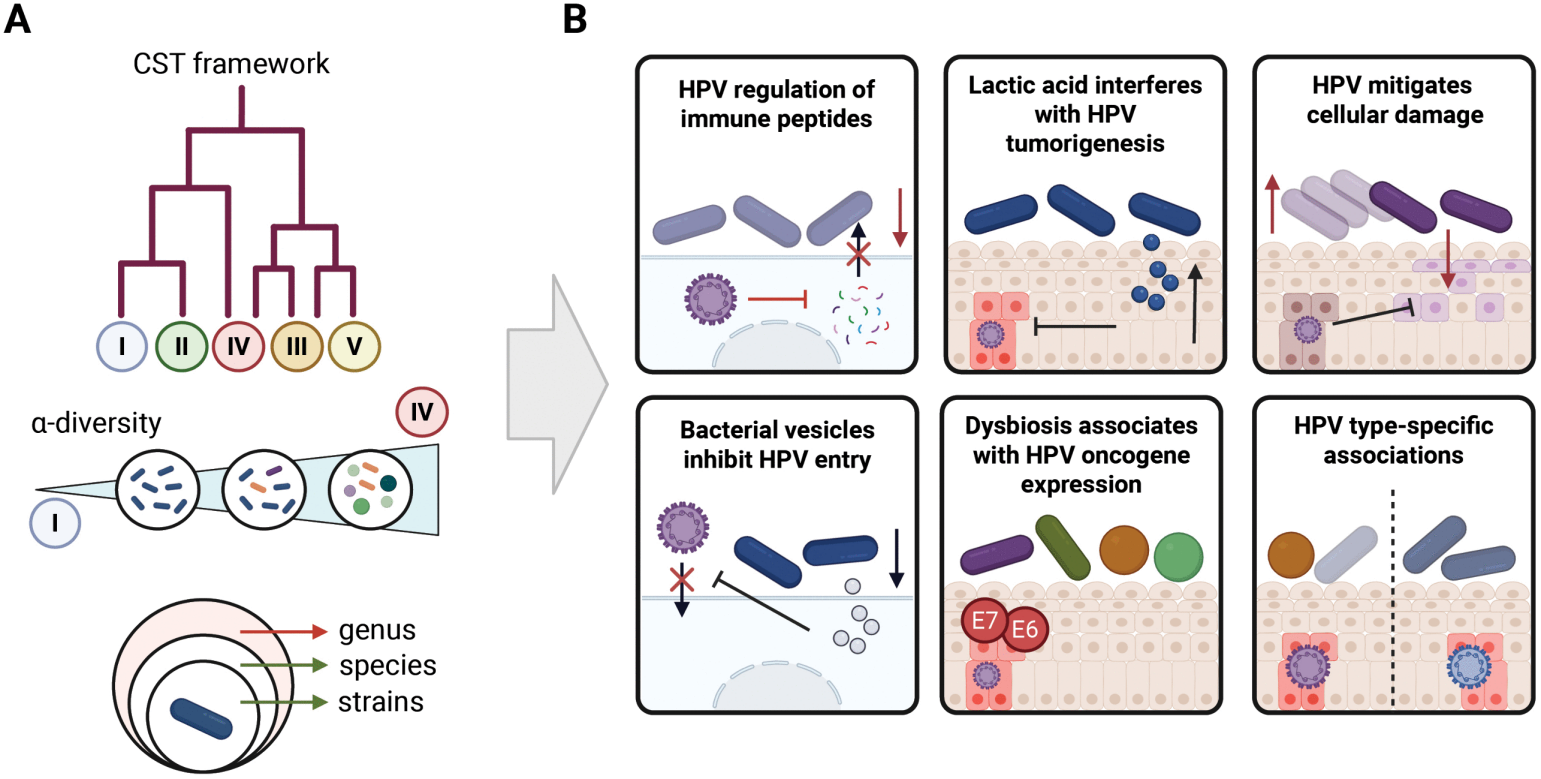
Community-level metrics overlook key mechanistic interactions between HPV and the cervicovaginal microbiome. **A**. Common analytical frameworks, including CST classification, α-diversity, and genus-level taxonomic summaries, offer only broad compositional overviews of the cervicovaginal microbiome. These approaches collapse species- and strain-level variation into simplified categories that do not capture biologically meaningful interactions. Red arrows indicate low-resolution, higher-level taxonomic summaries (e.g., genus), whereas green arrows indicate increasing biological resolution at the species and strain levels. **B**. Mechanistic studies demonstrate a much deeper layer of host–microbe–virus biology. HPV16 suppresses epithelial immune peptides, lactic acid isomers influence signaling pathways involved in HPV-driven transformation, HPV16 mitigates *Sneathia*-induced cytotoxicity, extracellular vesicles from *Lactobacillus crispatus* promote barrier repair and inhibit HPV16 entry, dysbiosis correlates with increased HPV oncogene expression, and distinct HPV genotypes associate with specific microbial functional profiles. Created in BioRender. Molina Beitia, M. (2025) https://BioRender.com/rlq28c6.

The prospect of identifying microbial biomarkers capable of refining cervical cancer screening or informing new therapeutic strategies has driven a surge of cross-sectional and longitudinal studies over the past decade [5,7–11]. Still, despite this intense activity, the literature remains surprisingly contradictory. Some studies report strong links between microbial composition and HPV outcomes [12–14], while others conclude inconsistent evidence or that no meaningful associations exist [15–19]. These conflicting interpretations persist even as mechanistic and multi-omic evidence increasingly clarifies how specific microbial functions shape epithelial immunity, viral oncogene expression, and early neoplastic changes (**Fig 1**) [20]. The field therefore finds itself in an unusual position: our mechanistic understanding is advancing rapidly, but our association studies are still dominated by outdated metrics and methodological choices that obscure rather than illuminate microbial–viral interactions.

A central source of confusion arises from the reliance on community state types (CSTs) and α-diversity as the primary descriptors of the cervicovaginal microbiome [21]. CSTs, although useful for early statistical work, were never intended to function as discrete biological categories [22,23]. Even in their expanded versions [24], CSTs flatten a highly dynamic, context-dependent microbial ecosystem into a handful of clusters. Recent work has shown that many women fall into intermediate or co-dominant states that do not align with CST definitions and that CSTs frequently fail to capture the functional and immunological differences between strains [25–27]. Similarly, α-diversity is often treated as an indicator of “dysbiosis,” despite being strongly influenced by geography, ancestry, menstrual cycle phase, sampling depth, sexual activity, and other contextual factors that confound, rather than reflect, HPV-specific biology [26–32]. These metrics are not capable of reflecting the mechanistic features that influence viral persistence or epithelial transformation and attempts to draw clinical conclusions from them inevitably lead to oversimplification (**Fig 1A**) [15,16,33].

Equally problematic are the statistical and ecological methods applied in many microbiome-HPV analyses. The application of LEfSe tools (linear discriminant analysis for differential abundance), the absence of proper multiple-testing correction, and the reliance on OTU clustering (the practice of collapsing similar 16S rRNA sequences into operational taxonomic units defined by fixed similarity cutoffs), all introduce biases that are well known in the microbiome field but remain remarkably common in cervicovaginal research [34–36]. These analytical choices inflate false positives, disguise meaningful within-person dynamics, and create artificial distinctions between groups [37–40]. Low-abundance or environmentally implausible taxa are sometimes interpreted as clinically significant biomarkers despite being likely artifacts of misclassification or contamination [41–43]. Similarly, many microbiome-HPV studies still rely on genus-level associations [44–48], nonetheless, genera often include species and strains with fundamentally different metabolic capacities, immunomodulatory effects, and epithelial interactions [49,50]. As emerging shotgun metagenomics studies make clear, clinically meaningful variation occurs at the strain and functional level [51–53], not at the genus level, underscoring the need for higher-resolution approaches to understand how specific microbial lineages shape HPV persistence and neoplastic progression [54–56]. Such patterns may indicate a deeper issue: statistical approaches are often applied without consideration of their assumptions, and ecological tools are used as if the cervicovaginal microbiome behaves like microbiomes from other anatomical sites.

These methodological shortcomings contrast sharply with the complexity of recent mechanistic work. Several high-impact studies now demonstrate that HPV actively reshapes the cervicovaginal microenvironment (**Fig 1B**). HPV16 has been shown to downregulate innate defense peptides essential for *Lactobacillus* metabolism, facilitating a shift toward anaerobic overgrowth and mucosal susceptibility [20]. Others have demonstrated that distinct lactic acid isomers produced by *Lactobacillus* species differentially regulate cervical epithelial stem cell renewal through YAP1 and PI3K-AKT signaling, revealing a direct microbial influence on the earliest stages of neoplastic transformation [57]. Recent work also shows that HPV16 directly alters epithelial responses to key vaginal bacteria such as *Sneathia*, enabling greater bacterial survival and mitigating toxin-induced damage (**Fig 1B**) [58]. Likewise, others demonstrate that *Lactobacillus crispatus* exerts direct antiviral and barrier-protective effects through its extracellular vesicles [59,60], which enhance epithelial repair, modulate macrophage polarization, and inhibit HPV16 entry [60]. Clinical studies integrating viral gene expression with cytokine and microbial profiles further show clear links between dysbiosis, mucosal inflammation, and high levels of HPV E6/E7 across preneoplastic lesions [61]. Metagenome-assembled genome-based studies now show HPV16/18 associate with distinct functional profiles on the vaginal microbiome, reinforcing that viral genotype matters (**Fig 1B**) [62]. Together, these findings provide a mechanistic and immunological framework that is far richer and more nuanced than the CST and diversity paradigm suggests.

Geographical and population-level variation further complicates interpretation. *Lactobacillus*-depleted vaginal communities are common and often stable in many African, Latin American, and Asian populations [19,29,63,64]. Treating such communities as inherently “dysbiotic” or “unhealthy” reflects a Western conceptual bias and leads to erroneous assumptions about risk. Without accounting for regional differences, HPV genotype distribution, sexual behavior, and access to healthcare, cross-sectional comparisons are likely to attribute population structure to “dysbiosis” or “microbial risk factors.” This misalignment contributes to inconsistent results across studies and restricts the generalizability of proposed biomarkers.

The path ahead requires bridging mechanistic understanding with rigorous ecological and statistical approaches. Adopting amplicon sequence variants-16S based workflows and contamination-aware pipelines is critical to avoiding the false detection of non-resident or ecologically implausible species [65,66]. Modern microbiome methods, paired with negative controls, prevalence filtering, and validated reference databases, are necessary to distinguish true cervicovaginal residents from reagent contaminants (e.g., kitome) or low-biomass artifacts [53,62,67–69]. Studies must also move beyond CSTs toward functional characterizations that reflect real microbial activity, including metabolomics, metatranscriptomics, and strain-level genomics [70–75]. HPV measurements should include viral load, E6/E7 expression, and integration status rather than DNA or genotype presence alone [3,76]. Longitudinal designs must replace single time points, and statistical frameworks capable of modeling within-person change [27,33,77–79], multi-pathogen interactions [15,80], and confounding is essential. Equally important is the inclusion of diverse populations to contextualize microbial patterns within global ecological variation [29].

The cervicovaginal microbiome is deeply intertwined with HPV biology, but the field has not yet adapted its methodological practices to match modern insights. If we are to translate microbiome knowledge into clinically useful tools [81,82], we must abandon oversimplified metrics and adopt approaches that reflect the true biological and ecological complexity of the host-microbe-virus interface—a transition that is not only overdue, but essential.
